# BALB/c Mice resist infection with *Bartonella bacilliformis*

**DOI:** 10.1186/1756-0500-1-103

**Published:** 2008-10-28

**Authors:** Beronica Infante, Sandra Villar, Sandra Palma, Jenny Merello, Roberto Valencia, Luis Torres, Jamie Cok, Palmira Ventosilla, Ciro Manguiña, Humberto Guerra, Cesar Henriquez

**Affiliations:** 1 Instituto de Medicina Tropical Alexander von Humboldt – Universidad Peruana Cayetano Heredia, Lima, Perú; 2 Departamento de Enfermedades Transmisibles y Dermatológicas, Hospital Nacional Cayetano Heredia, Lima, Perú; 3 Facultad de Medicina Veterinaria y Zootecnia – Universidad Peruana Cayetano Heredia, Lima, Perú; 4 Departamento de Anatomia Patológica, Hospital Nacional Cayetano Heredia, Lima, Perú

## Abstract

**Background:**

Bartonellosis due to *Bartonella bacilliformis *is a highly lethal endemic and sometimes epidemic infectious disease in South America, and a serious public health concern in Perú. There is limited information on the immunologic response to *B. bacilliformis *infection. The objective of this research was to produce experimental infection of BALB/c mice to *B. bacilliformis *inoculation.

**Findings:**

BALB/c mice were inoculated with 1.5, 3.0 or 4.5 × 10^8 ^live *B. bacilliformis *using different routes: intraperitoneal, intradermal, intranasal, and subcutaneous. Cultures of spleen, liver, and lymph nodes from one to 145 days yielded no cultivable organisms. No organs showed lesions at any time. Previously inoculated mice showed no changes in the reinoculation site.

**Conclusion:**

Parenteral inoculation of live *B. bacilliformis *via different infection routes produced no macroscopic or microscopic organ lesions in BALB/c mice. It was not possible to isolate *B. bacilliformis *using Columbia blood agar from 1 to 15 days after inoculation.

## Background

Carrion's disease is an infectious disease; endemic in some regions of Peru, Colombia and Ecuador [1]. The etiologic agent is *Bartonella bacilliformis*. In endemic areas, the incidence of infection has been estimated at 12.7/100 person-years [2]. Carrion's disease is present in the interandean valleys located between 500 and 3200 m above sea level [3,4], where ecological conditions are favorable for sand flies of the genus *Lutzomyia*, some species of which transmit the disease [5]. No reservoirs other than humans have been implicated in endemic areas[6-9].

There is limited information on the immunologic response to *Bartonella *infection but it is widely known that antibodies are detectable in convalescent patients with long-term protective immunity [10-12]. The presence of chronic asymptomatic carriers in endemic areas and the appearance of the chronic phase contribute to the speculation that innate immunity and humoral immunity may not be completely effective in clearing *Bartonella *infection.

The most studied immunological model related to Carrion's disease is *B. henselae *infection, which results in a wide range of clinical conditions including Cat Scratch Disease (CSD) [13-15]. There is no good animal model for *B. bacilliformis *infection [16]. Sensitized rabbits with viable *B. bacilliformis *and increased their susceptibility to lethality of subsequently administered *Bartonella *metabolites. The objective of this research was to produce experimental infection of BALB/c mice to *B. bacilliformis *inoculation.

## Methods

### Bacterial strain

A single *B. bacilliformis *strain (ATCC#35685) and a recently isolated strain from an acute phase patient (IMTAVH#00032) was used in this study. *B. bacilliformis *stocks were grown on 10% sheep blood Columbia Agar flasks at 29°C. Bacteria were harvested in a laminar flow hood, scraping colonies off the agar surface into brain heart infusion (BHI) broth media. The cells were collected by centrifugation and suspended in phosphate-buffered saline solution (PBS). Colony-forming units (CFU) of harvested *Bartonella *cultures were estimated using the Mc Farland turbidity scale (0.5 mL of tube 0.5 of Mc Farland scale ~1.5 × 10^8 ^CFU/mL).

### Inoculations

*B. bacilliformis *was prepared as described above, and thawed on ice prior to inoculation. Forty six BALB/c mice (10–12 weeks old) were anaesthetized with ketamine and administered live *B. bacilliformis *by the routes indicated as described below. Additionally, forty six BALB/c control mice were inoculated with sterile saline.

For intraperitoneal (i.p.), intradermal (i.d.) and subcutaneous (s.c.) immunization, mice were dosed with volumes of 200 – 500 μL containing around 1.5, 3, or 4.5 × 10^8 ^CFU of *B. bacilliformis *administered using a tuberculin syringe and needle. For oral immunizations, mice were dosed by using a 22-gauge gavages (force feeding) needle attached to a 1 mL syringe. For intranasal (i.n.) immunizations, mice were dosed with volumes of 10–50 μL containing around 1.5 × 10^8 ^CFU of *B. bacilliformis *administered into the nostrils using a micropipette fitted with a 200 μL tip. Following immunizations, mice were observed daily for signs of illness.

Animals were sacrificed at different times up to day 60 post-infection. Sections of formalin-fixed liver, spleen, lung, kidney, brain, and lymph node tissue were stained with hematoxylin and eosin.

### Detection of *B. bacilliformis *in vivo

From all mice, bacterial loads in liver, spleen, lung, kidney, brain and lymph nodes were determined by plating of 10-fold serial dilutions of organ homogenates on Columbia sheep blood agar. In addition, quantitative cultures of blood specimens were performed at the time of necropsy. Before samples were plated on Columbia agar, erythrocytes were lysed either by freezing and thawing or by vigorous mixing with distilled water. All cultures were incubated at 27°C for at least 4 weeks before being recorded as negative for growth.

Additionally, 4 i.d. immunized mice were subjected to intradermal skin testing with *B. bacilliformis *(using the harvested ATCC#35685 strain in 2 mice and the harvested IMTAVH#00032 strain in the other 2), to monitor delayed swelling reactions as an indication of specific immune induction. The mice were boosted i.d. with 3 × 10^8 ^CFU on day 50 after primary i.d. inoculation. One week after the boost, each mouse was challenged in the right dorsal skin with the same number of bacteria. The injection site was monitored at 24, 48, and 72 hours for swelling responses and compared to the control skin as an external negative control. Swelling was determined from three readings using a caliper.

## Results

Eleven immunizations were done using different doses of *B. bacilliformis *at different incubation periods (Table 1).

**Table 1:** Experiments attempting infection of BALB/c mice with viable *Bartonella bacilliformis*

**Exp**	**Route**	**No. of Mice**^**a**^	**CFU**^**b**^	**Time to sacrifice**	**Results of H/E stain**
1	i.p.	03	1.5 × 10^8^	7 days	organs normal
2	i.p.	05	1.5 × 10^8^	6, 24 hs & 14 days	organs normal
3	i.p.	05	1.5 × 10^8^	6, 24 hs & 14 days	organs normal
4	s.c.	03	4.5 × 10^8^	24 hs	skin normal
5	s.c.	02	4.5 × 10^8^	30 days	skin normal
6	s.c.	07	1.5 × 10^8^	2, 6 & 8 hs	skin normal
7	Oral	03	1.5 × 10^8^	24 hs	organs normal
8	Oral	05	1.5 × 10^8^	24 hs	organs normal
9	i.n.	04	1.5 × 10^8^	24 hs	organs normal
10	i.d.	03	4.5 × 10^8^	7 days	skin normal
11	i.d.	02	4.5 × 10^8^	30 days	skin normal
12^b^	i.d.	02	3 × 10^8^	24, 48 & 72 hs	No skin changes
13^c^	i.d.	02	3 × 10^8^	24, 48 & 72 hs	No skin changes

^a^Forty six control mice inoculated with sterile saline

^b^previously inoculated with viable ATCC#35685

^c^previously inoculated with viable, recently isolated *B. bacilliformis *from an acute phase patient

Spleen, liver, brain, and lymph nodes cultures did not show cultivable organisms within the first 6 hours, 24 hours, and 7–14 days of infection. No organisms could be recovered from blood or skin. Kidneys and lungs remained sterile throughout this experiment. Subsequent experiments with inoculate ranging from 1.5, 3, or 4.5 × 10^8 ^CFU yielded similar results.

One animal showed histopathological alterations, 24 hours after i.p. inoculation of 1.5 × 10^8 ^viable *B. bacilliformis*. Many abscesses were found in the liver of this mouse. Bacterial cultures were negative. Control mice were negative.

No other organs showed abscess at any time. No granulomatous lesions were found in hematoxylin and eosin stained liver sections.

Mice previously inoculated with live *B. bacilliformis *showed no change in the site of a second inoculation (dorsal skin).

## Discussion

We have performed different mouse inoculation experiments with varying numbers of *B. bacilliformis*. No bacteria were recovered from organs of inoculated mice. The Columbia Blood agar has been used to isolate *B. bacilliformis *in the clinical practice and is the medium for growing our bacteria in experimental conditions. In our study the isolation of bacteria was not successful even when using high inocula. Factors involved in host specificity and ligand-receptor affinity can be involved in failure to reproduce infection by many *Bartonella *species in BALB/c mice [17]. Thin blood films were negative in our experiments; thus, there was no bacteremia in BALB/c mice at any time or by any inoculation route.

Intradermal and subcutaneous immunizations are the experimental routes of infection most similar to natural human infection, which is due to inoculation by the *Lutzomyia *bite. Our first experiments using the intradermal route do not show histopathological lesions in skin nor systemic infection even using higher inocula. Previously immunized mice with harvested ATCC#35685 strain have not shown any delayed-type hypersensitivity when they were exposed to the same strain 50 days later.

A recently isolated (supposedly actively pathogenic) strain from an acute phase patient was incapable of eliciting an intradermal immune response. Boosters after 50 days have not been useful to induce a cellular immune response using harvested bacteria. Karem *et al *[14] showed that two inoculations of *B. henselae *were enough to show a cellular immune response in BALB/c mice. Many boosters with *B. bacilliformis *may be necessary to induce a cellular response.

We have found only one animal with liver abscesses 24 hours post-inoculation. The sterile saline inoculated (control) mouse showed similar liver abscesses, probably attributed to a disease different to Bartonella.

## Conclusion

The results of our experiments show that *B. bacilliformis *didn't produce macro and microscopic organ lesions or disease in BALB/c mice, using different inoculation routes, and that bacteremia was absent after one to 15 days after inoculation.

Intradermal re-inoculation of harvested *B. bacilliformis *didn't elicit delayed-type hypersensitivity in BALB/c mice even after a booster dose at 50 days of the primary inoculation.

BALB/c mice are not useful as models for the human *B. bacilliformis *infection, but exploration of their apparent resistance to infection may yield important immunological insights. New experiments to detect cytokine, immunoglobulin and cellular responses are necessary to develop these explorations.

## Authors' contributions

IB carried out the inoculations, drafted the manuscript, and participated in microbiology studies. VS carried out the inoculations. PS carried out the inoculations. MJ participated in microbiology studies. VR participated in the inoculations, and veterinary assistance, TL participated in the inoculations, and veterinary assistance, CK participated in pathology studies, VP participated in inoculations, microbiology studies, and drafted the manuscript, CM participated in the design of the study, GH participated in the design of the study, microbiology studies, and drafted the manuscript, CH conceived of the study, and participated in its design and coordination and helped to draft the manuscript. All authors read and approved the final manuscript.

## Competing Interests

The authors declare no competing interests.
